# Supplementary material for: Characterization of Growth-Promoting Activities of Consortia of Chlorpyrifos Mineralizing Endophytic Bacteria Naturally Harboring in Rice Plants—A Potential Bio-Stimulant to Develop a Safe and Sustainable Agriculture
Source: Microorganisms. 2023 Jul 16;11(7):1821. doi: 10.3390/microorganisms11071821 (PMC10385066; doi:10.3390/microorganisms11071821)
Supplement: Supplementary file 1 [file microorganisms-11-01821-s001.zip › microorganisms-2475009-supplementary.pdf]

Table S1: Biodegradations profile of chlorpyrifos (1 gm/100 mL) in minimal broth medium after treated with endophytic bacterial synthetic Consortium-2.

Similarity of hit	Spectrum	Soft ionization (SI)	Spectrum	Molecular weight (Da)	Molecular form	Molecular structure
1	78	2921	88	2	Chlorpyrifos	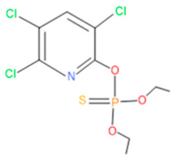
2	70	2921	88	2	Chlorpyrifos	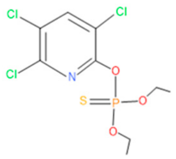
3	67	2921	88	2	Chlorpyrifos	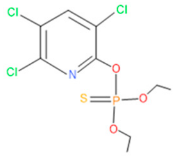
4	67	2921	88	2	Chlorpyrifos	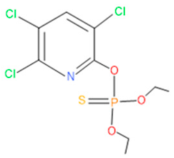
5	65	2921	88	2	Chlorpyrifos	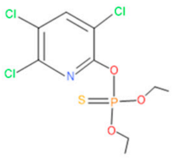
6	59	2588	3	6	Phorate sulfone	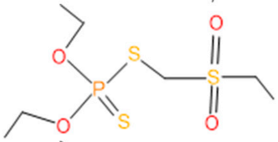
7	59	2588	3	6	Phorate sulfone	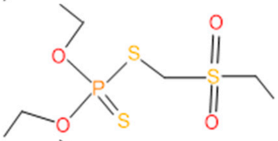
8	58	2588	4	7	Phorate sulfone	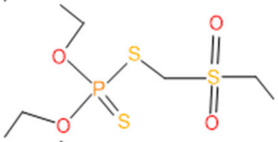
9	58	2588	3	6	Phorate sulfone	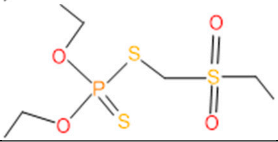

10	58	2921	88	2	Chlorpyrifos	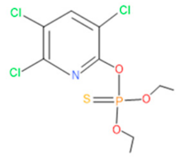
11	57	2588	4	7	Phorate sulfone	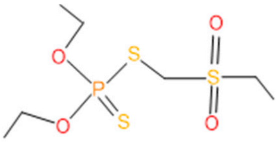
12	56	5598	13	0	Chloropyriphos-methyl	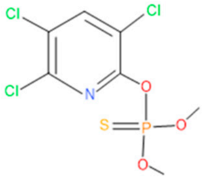
13	55	2921	88	2	Chlorpyrifos	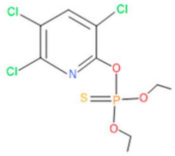
14	55	6515	38	4	2-Hydroxy-3,5,6-trichloropyridine	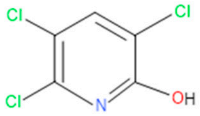
15	55	2588	4	7	Phorate sulfone	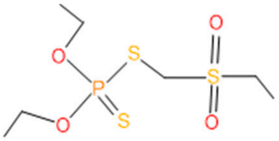
16	55	6515	38	4	2-Hydroxy-3,5,6-trichloropyridine	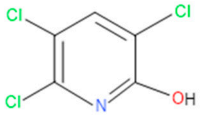
17	54	17297	40	4	Carbofenothion sulfoxide	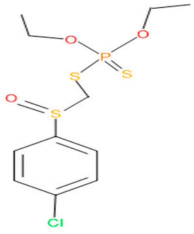
18	53	2588	4	7	Phorate sulfone	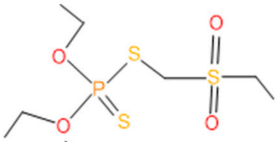
19	52	2497	7	6	Oxydisulfoton	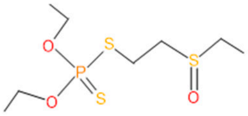

20	52	16947	69	6	Carbonochloridic acid	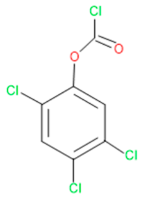
21	51	4891	54	7	Thionodemeton sulfone	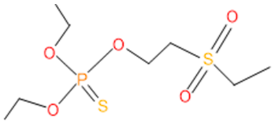
22	51	2021	58	1	dl-2-. beta. -Thienyl-. alpha. -alanine	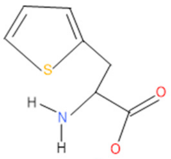
23	50	5598	15	2	Phosphoric acid, diethyl 3,5,6-trichloro-2-pyridyl ester/Chlorpyrifos Oxon	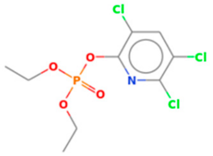
24	50	683	8	9	Diethyl methanephosphonate	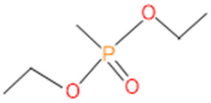
25	50	683	8	9	Diethyl methanephosphonate	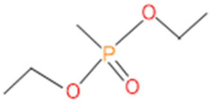

Table S2: Biodegradations profile of chlorpyrifos (1 gm/100 mL) in minimal broth medium after treated with endophytic bacterial synthetic Consortium-4.

Similarity of hit	search Spectrum	Soft ionization (SI)	Spectrum	Molecular weight (Da)	Molecular form	Molecular structure
1	78	2921	88	2	Chlorpyrifos	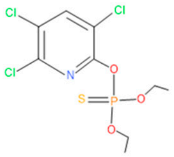
2	70	2921	88	2	Chlorpyrifos	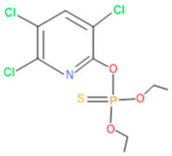
3	67	2921	88	2	Chlorpyrifos	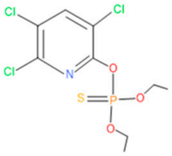
4	67	2921	88	2	Chlorpyrifos	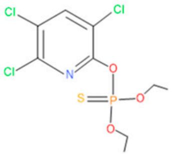
5	65	2921	88	2	Chlorpyrifos	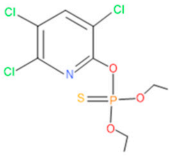
6	59	2921	88	2	Chlorpyrifos	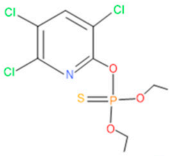
7	59	2588	3	6	Phorate sulfoxide	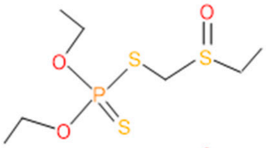
8	58	2588	3	6	Phorate sulfoxide	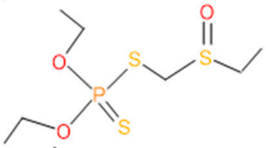
9	58	2588	4	7	Phorate sulfone	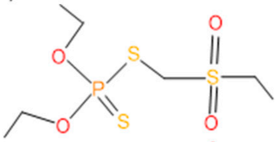
10	58	2588	3	6	Phorate sulfoxide	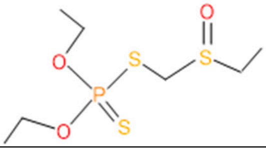

11	57	2588	4	7	Phorate sulfone	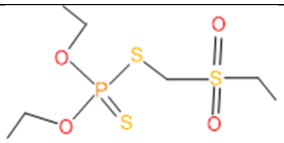
12	56	2921	88	2	Chlorpyrifos	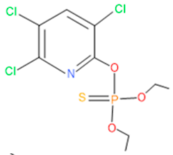
13	55	2588	4	7	Phorate sulfone	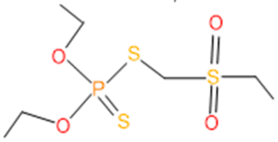
14	55	6515	38	4	2-Hydroxy-3,5,6-trichloropyridine	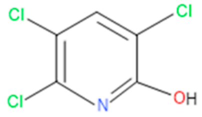
15	55	6515	38	4	2-Hydroxy-3,5,6-trichloropyridine	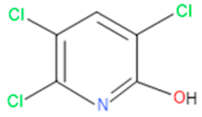
16	55	2588	4	7	Phorate sulfone	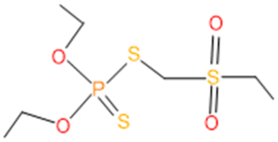
17	54	5598	13	0	Chloropyriphos-methyl	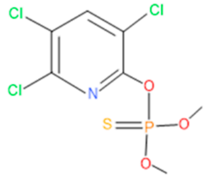
18	53	16947	69	6	Carbonochloridic acid	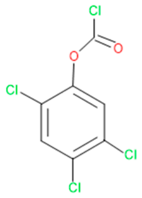
19	52	5598	15	2	Phosphoric acid	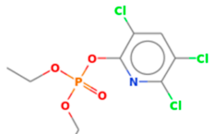
20	52	17297	40	4	Carbofenothion sulfoxide	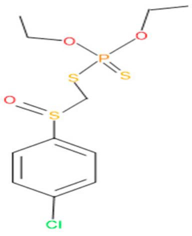

21	51	4891	54	7	Thionodemeton sulfone	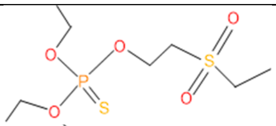
22	51	2497	7	6	Oxydisulfoton	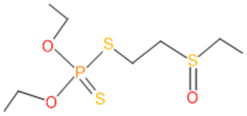
23	50	16270	87	4	Phosphorodithioic acid	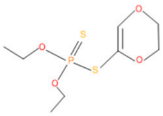
24	50	5598	13	0	Chloropyriphos-methyl	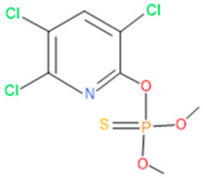
25	50	4861	59	0	Thiophene,	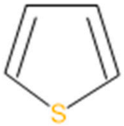

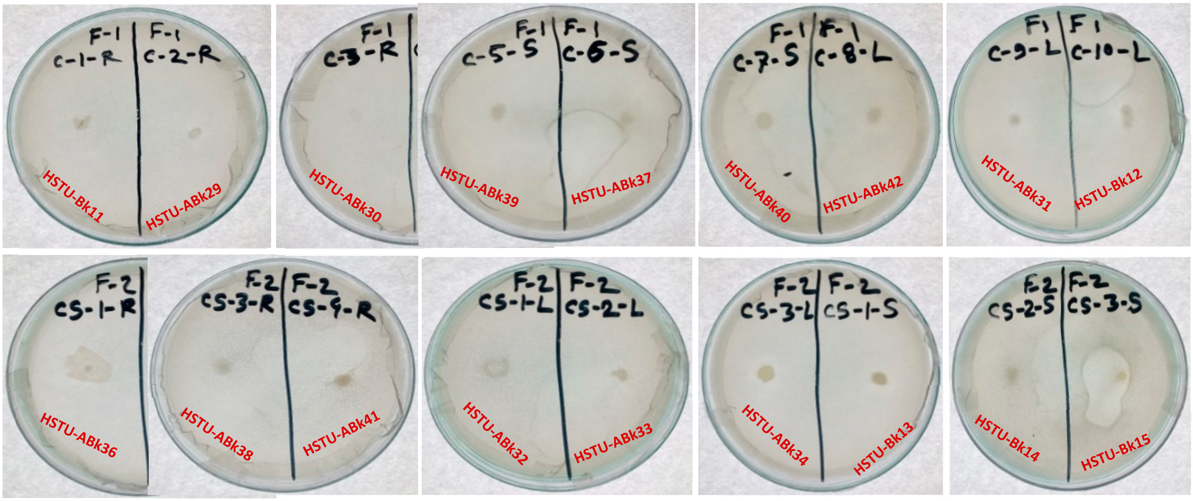

Figure S1: phosphate solubilizing activities of the chlorpyrifos mineralizing endophytic bacteria

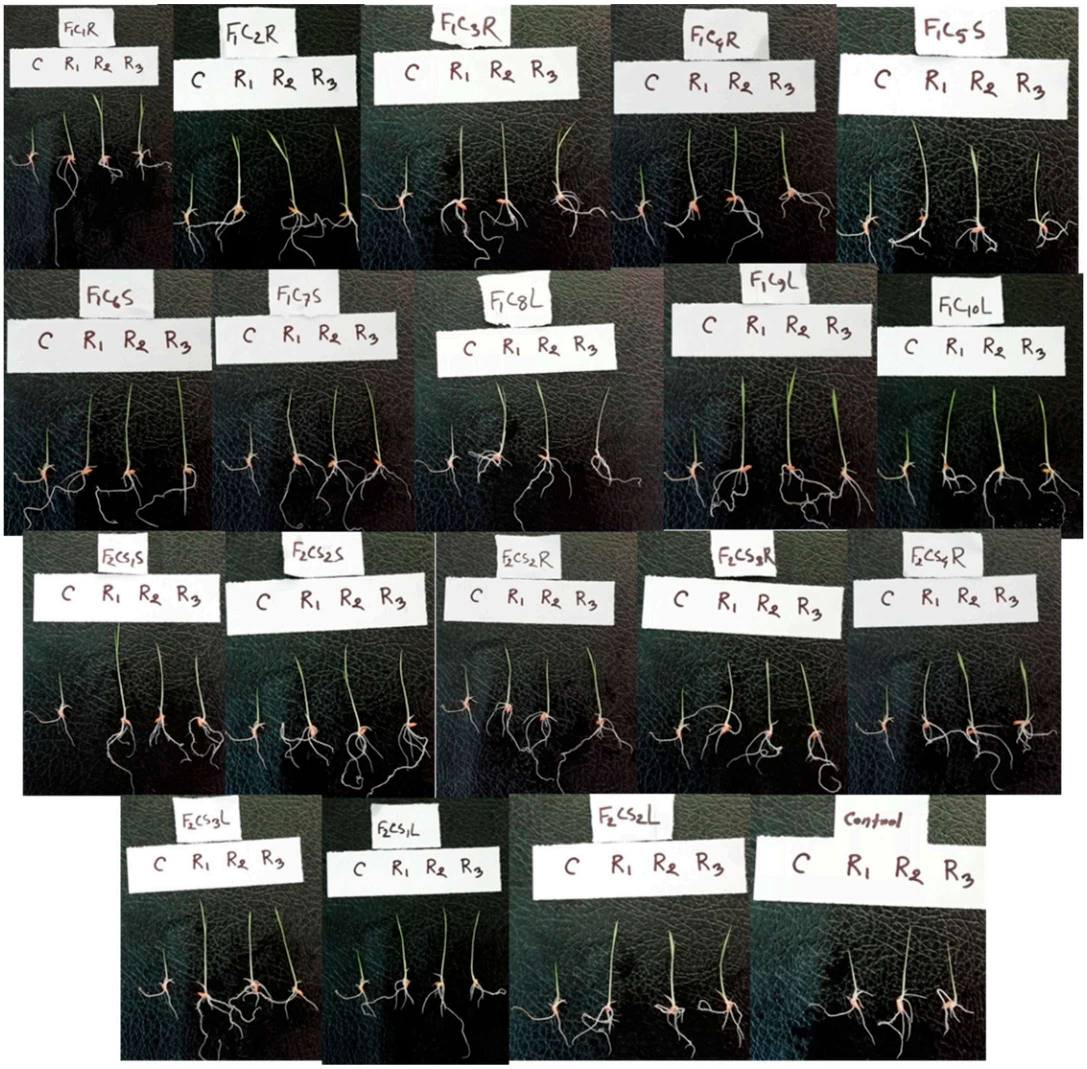

F1C1R
C R1 R2 R3
F1C2R
C R1 R2 R3
F1C3R
C R1 R2 R3
F1C4R
C R1 R2 R3
F1C5S
C R1 R2 R3
F1C6S
C R1 R2 R3
F1C7S
C R1 R2 R3
F1C8L
C R1 R2 R3
F1C9L
C R1 R2 R3
F1C10L
C R1 R2 R3
F2CS1S
C R1 R2 R3
F2CS2S
C R1 R2 R3
F2CS2R
C R1 R2 R3
F2CS3R
C R1 R2 R3
F2CS4R
C R1 R2 R3
F2CS3L
C R1 R2 R3
F2CS1L
C R1 R2 R3
F2CS2L
C R1 R2 R3
Control
C R1 R2 R3

(a)

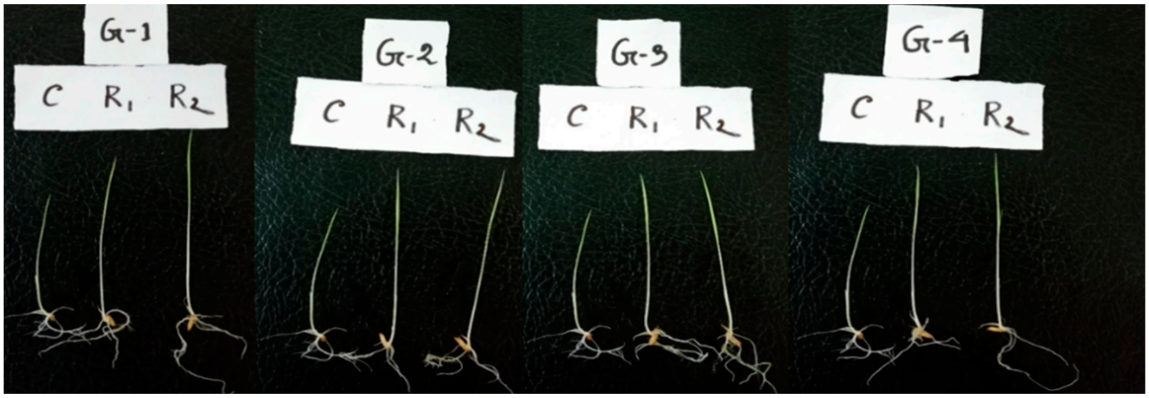

G-1
C R1 R2
G-2
C R1 R2
G-3
C R1 R2
G-4
C R1 R2

(b)

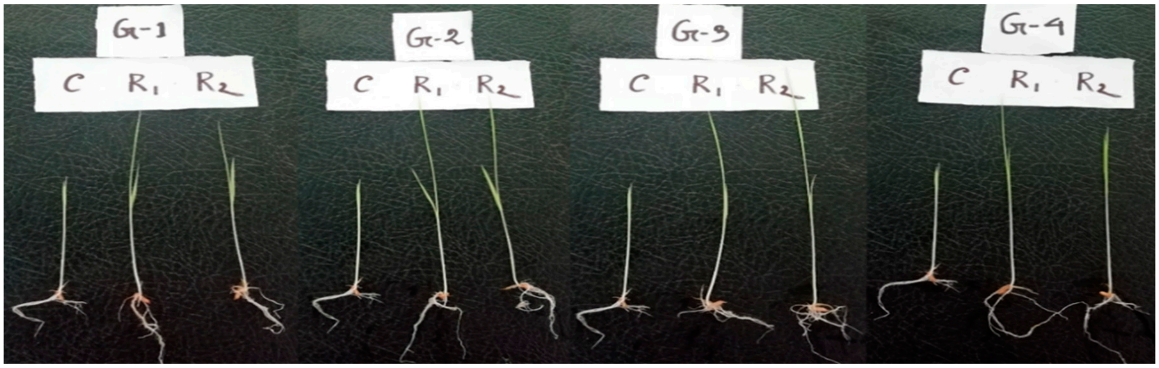

(c)

Figure S2: (a) Germinated rice plants after 8 days of endophytic bacterial treatment. (C) control, (R) replica; (b) Effect of endophytic consortium on seedling and growth parameter after 8 days. (C) control, (R) replica and (G) consortia; (c) Effect of endophytic consortium on Seedling and growth parameter after 12 days of treatment. (C) control, (R) replica and (G) consortia.

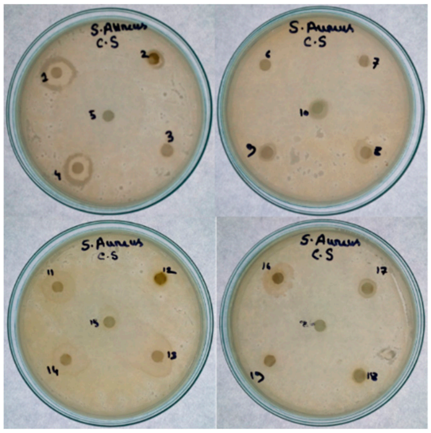

(a)

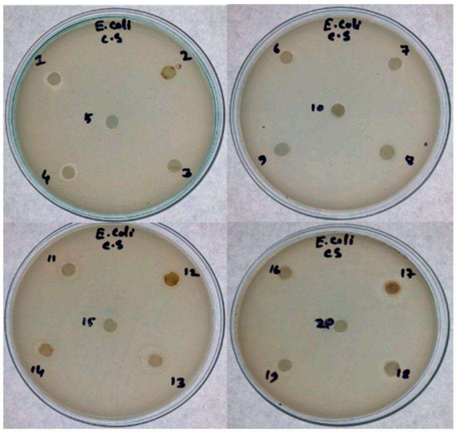

(b)

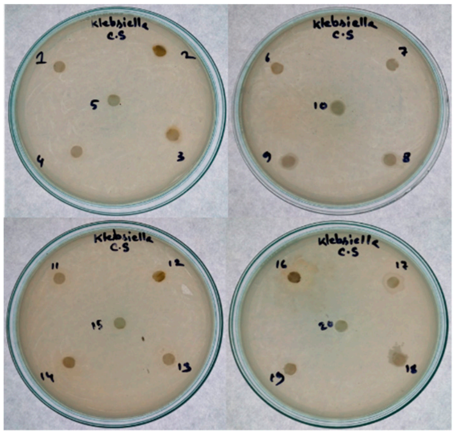

(c)

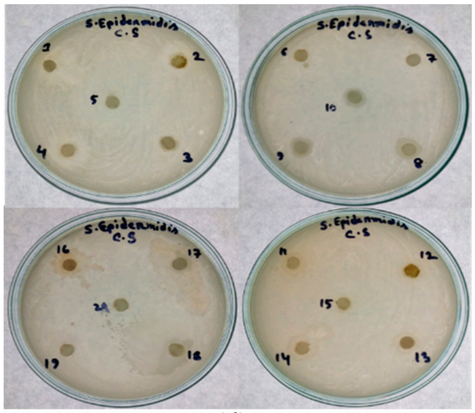

(d)

Figure S3: (a) Antibacterial activity of endophytic bacteria against multidrug-resistant human pathogenic bacteria *S. Aureus* after 32 h. (b) Antibacterial activity of endophytic bacterias against multidrug-resistant human pathogenic bacteria *E. Coli* after 32 h. (c) Antibacterial activity of endophytic bacteria against multidrug-resistant human pathogenic bacteria *Klebsiella* sp. after 32 h. (d) Antibacterial activity of endophytic bacteria against multidrug-resistant human pathogenic bacteria *S. epidermidis* after 32 h.
